# A novel built-in adjuvant metallothionein-3 aids protein antigens to induce rapid, robust, and durable immune responses

**DOI:** 10.3389/fimmu.2022.1024437

**Published:** 2022-11-08

**Authors:** Ying Yin, Yanfei Gu, Xiaodong Zai, Ruihua Li, Xinjie Zhu, Rui Yu, Jun Zhang, Shuyi Wang, Yue Zhang, Jian Lin, Junjie Xu, Wei Chen

**Affiliations:** ^1^ Laboratory of Vaccine and Antibody Engineering, Beijing Institute of Biotechnology, Beijing, China; ^2^ Synthetic and Functional Biomolecules Center, Peking University, Beijing, China; ^3^ Department of Pharmacy, Peking University Third Hospital, Beijing, China

**Keywords:** metallothionein-3, adjuvants, vaccines, protein antigens, immune responses

## Abstract

Adjuvants are crucial components of vaccines that can enhance and modulate antigen-specific immune responses. Herein, we reported for the first time that human metallothionein-3 (MT3), a low molecular weight cysteine-rich metal-binding protein, was a novel promising adjuvant candidate that could help protein antigens to induce rapid, effective, and durable antigen-specific immune responses. In the present study, MT3 was fused to outer membrane protein 19 (Omp19) of *Brucella abortus* (MT3-Omp19, MO) and C fragment heavy chain (Hc) of tetanus neurotoxin (MT3-Hc, MH), respectively. The results showed that MT3 as a built-in adjuvant increased the Omp19- or Hc-specific antibody responses by 100-1000 folds in seven days after primary immunization. Compared to other commercially available adjuvants, MT3 could stimulate earlier (4 days after primary injection) and stronger (10-100 folds) antibody response with lower antigen dose, and its adjuvanticity relied on fusion to antigen. Although the mechanism was not clear yet, the fusion protein MO was observed to directly activate DCs, promote germinal center formation and improve the speed of Ig class switching. Interestingly, our subsequent study found that other members of the mammalian MT family (human MT1 or murine MT3 for examples) also had potential adjuvant effects, but their effects were lower than human MT3. Overall, this study explored a new function of human MT3 as a novel built-in adjuvant, which may have important clinical application potential in vaccine development against global pandemics.

## Introduction

Vaccines are effective tools to prevent and control infectious diseases. As the crucial components of vaccines, adjuvants could enhance and modulate antigen-specific immune responses by triggering and regulating the innate and acquired immunity (1). Several adjuvants have been approved in licensed vaccines for clinical use, such as aluminum salt, MF59, AS0 system and CpG1018 (2, 3). With the continuous progress of vaccinology and basic immunology, the researches on new adjuvants were also diversified. Adjuvants that induce CD8^+^ T cells and tissue resident memory T cells (4), adjuvants that target non-Toll-like receptor (TLR) pattern recognition receptors, metabolic adjuvants, cell death adjuvants and epigenetic adjuvants were all the frontiers in adjuvants design and development (5).

Metallothioneins (MTs) are a family of cysteine-rich metal-binding proteins with a low molecular weight about 7 kDa. It was first discovered in studying the accumulation of metal cadmium in horse kidney (6). MTs are broadly distributed in all kinds of species, including animal, plant, microorganism, and human (6, 7). There are four subfamilies of MTs in human, named MT1, MT2, MT3 and MT4 (8). It was reported that MTs could regulate the homeostasis of copper and zinc, mitigate heavy metal poisoning, and alleviate superoxide stress (7). The *in vivo* distributions of distinct MTs are different. MT3 can maintain intracellular metal homeostasis, protect cells from oxidative stress, and regulate cell growth and differentiation like other MTs, but it is mainly expressed in the central nervous system, and is supposed to have a unique neuronal growth inhibitory activity (8–10). It has been reported that the common structure among human MTs includes a preserved 20 cysteine residues and two major domains, which wrap around a metal-thiolate cluster known as the α (C-terminal)- and β (N-terminal)-domain (11). However, MT3 has several unique structure features, which is absent in the structure of other subfamilies of MTs. At the N terminal of its β domain, there are a conserved “TCPCP” sequence and an acid-basic catalysis motif “KCE”, and at the C terminal of its α domain, there are a “EAAEAE” hexapeptide insertion (11). This indicated that human MT3 may be partially functionally different from other MTs. It has been designed as a component of chemotherapy drug delivery carrier for anti-tumor therapy previously (12).

In this research, we reported for the first time that human MT3 is a promising build-in adjuvant that could help protein antigens to induce rapid, robust, and durable immune responses. As a proof of concept, MT3 was fused to outer membrane protein 19 (Omp19) of *Brucella abortus* (MT3-Omp19, MO) and C fragment heavy chain (Hc) of tetanus neurotoxin (MT3-Hc, MH), respectively. *Brucella* is a facultative intracellular Gram-negative bacterial pathogen, and Omp19 was regarded as a protective antigen for development of brucellosis candidate vaccine (13, 14). Tetanus toxin is a neurotoxin produced by the Gram-positive bacterium *Clostridium tetani*, and Hc retains full binding affinities to neuronal cells *via* gangliosides in lipid rafts and is an important antigen to vaccine development against tetanus (15–17). The results showed that MT3 could increase the Omp19- or Hc-specific antibody responses by 100-1000 folds in seven days after primary immunization, and stimulate earlier and stronger antibody response with lower antigen dose compared to other commercially available adjuvants. Our study suggested that human MT3 may have important clinical application potential in vaccine development against global pandemics.

## Materials and methods

### Preparation of immunogens

MT3 (human)-Omp19 (MO), MT1 (human)-Omp19 (M_1_O), MT3 (murine)-Omp19 (M_m_O), MT3 (human)-Hc (MH) and MT3 (human) genes were codon-optimized for expression and synthesized. The proteins were expressed from a pET28a plasmid in *E. coli* BL21. The fusion proteins were purified from bacterial lysate supernatants by a HisTrap™ affinity chromatography column (Cytiva, Uppsala, Sweden) and DEAE Sepharose™ Fast Flow column (Cytiva, Uppsala, Sweden). The effluents corresponding to the fusion proteins were treated with an endotoxin removing gel (Cytiva, Uppsala, Sweden). The fusion proteins (MO, M_1_O, M_m_O, MH, and MT3) were collected, and assessed with SDS-PAGE and Western blot analysis. In Western blot analysis, the bands were probed with Omp19 immunized mice serum and goat anti-mouse IgG Fc (HRP) (Abcam, ab97265, Cambridge, UK) against Omp19, MO, M_1_O and M_m_O; with human monoclonal antibody TT0067 and goat anti-human IgG Fc (HRP) (Abcam, ab97225, Cambridge, UK) against Hc and MH, respectively. Hc and Omp19 were prepared as described previously (18, 19). Lipopolysaccharide (LPS) contamination was identified by the tachyplens amebocyte lysate (TAL) assay (Chinese Horseshoe Crab Reagent Manufactory, Xiamen, China).

### Preparation of other adjuvants

According to the manufacturer’s instructions, antigens were mixed or adsorbed with the different commercial adjuvants for vaccine preparation. Briefly, Pam3CSK4 (VacciGrade™, 10 μg), Poly (I:C) (HMW, VacciGrade™, 10 μg), MPLA-SM (VacciGrade™, 10 μg), R848 (VacciGrade™, 10 μg), and Quil-A^®^ Adjuvant (5 μg) were mixed with 5 μg of Omp19 per mouse in phosphate buffer saline (PBS), respectively. AddaS03™ or AddaVax™ was added to 5 μg Omp19 suspension at a final volume ratio of 1:1. Alhydrogel^®^ Alum adjuvant (Al, 50 µg) (Croda Denmark, Frederikssund, Denmark) was added to the 5 μg Omp19 suspension per mouse; and CpG (25 µg, synthesized by TAKARA BIO INC, Dalian, China) was added to the Omp19 plus Alum adjuvant suspension (25 μg) per mouse. Except Al and CpG, all the other commercial adjuvants were purchased from In vivoGen (Toulouse, France).

### Immunization, sampling schedule and ethics statements

Female BALB/c mice (6–8 weeks) were purchased from Beijing SPF Biotechnology Co. Ltd. (Beijing, China). The animals were bred under specific pathogen-free conditions. The experiments involving animals were carried out according to the Institutional Experimental Animal Welfare and Ethics Committee guidelines. Mice were immunized with antigen or antigen plus adjuvant at final volumes of 100 μL by intramuscular (i.m.) injection. Animals were vaccinated in accordance with different immunization schedules: either a single injection or a standard schedule consisting of a primary injection on day 0 and a booster on day 14. Sera were collected at the indicated days post-immunization.

### Enzyme-linked immunosorbent assay (ELISA)

Antigen-specific serum antibodies (including IgG, IgG1 and IgG2a, IgM) were determined by ELISA. Serum samples were serially diluted in 0.1% bovine serum albumin (BSA)/phosphate buffer saline- Tween-20 (PBST) and incubated for 1 h at 37°C. After washing, horseradish peroxidase (HRP)-conjugated goat anti-mouse IgG (Abcam, Cambridge, UK), IgG1, IgG2a and IgM (Santa Cruz, Dallas, USA) were added to the samples and incubated for 1 h at 37°C. Tetramethylbenzidine (TMB) single-component substrate solution and stop solution (Solarbio, Beijing, China) were added, and the assay was then measured at 450 nm/630 nm (SPECTRA MAX 190, Molecular Device, USA). The titers were presented as the highest sample dilution resulting in an optical density (OD) value greater than 2.1 times the mean of the OD of the nonimmune control serum.

### Dendritic cell (DC) activation and flow cytometric analysis

Bone marrow-derived DCs (BMDCs) were generated by cultivating bone marrow cells in RPMI 1640 (Thermo Scientific, Waltham, USA) supplemented with 10% fetal bovine serum (FBS) (Thermo Scientific, Waltham, USA), 1% Penicillin/Streptomycin solution (Sigma-Aldrich, City of Saint Louis, USA), 20 ng/mL mouse granulocyte-macrophage colony-stimulating factor (mGM-CSF) (R&D, Minneapolis, USA) and 10 ng/mL mouse interleukin-4 (mIL-4) (R&D, Minneapolis, USA) for 7 days. The cells were stimulated with 10 μg/mL MO, 10 μg/mL Omp19, 0.1 μg/mL LPS (Sigma-Aldrich, City of Saint Louis, USA) or PBS for 24 h, respectively. The expression levels of CD11c (FITC Hamster anti-mouse CD11c, BD Pharmingen, clone HL3, Franklin Lakes, USA), MHC II (Alexa Fluor^®^ 647 Rat anti-mouse I-A/I-E, BD Pharmingen, clone M5/114.15.2, Franklin Lakes, USA), and CD80 (PE Hamster anti-mouse CD80, BD Pharmingen, clone 16-10A1, Franklin Lakes, USA) on DCs were evaluated by BD canto II (BD Biosciences, USA). The data were analyzed in FACS Diva™ Software.

### Early GC reactions and flow cytometric analysis

In order to avoid variations linked to sex (20, 21), female BALB/c mice aged 6-8 week were vaccinated at day 0 *via* i.m. route with MO (5μg), Omp19 (5μg), Omp19+Al+CpG (5µg+50µg+25µg), and PBS (MO, Omp19, Omp19+Al+CpG, n=5; PBS, n=3). Draining lymph nodes (DLNs) on day 4 were harvested to prepare single-cell suspensions. The single-cell suspensions were blocked with anti-mouse CD16/CD32 monoclonal antibody (BD Biosciences, Franklin Lakes, USA). The live/dead fixable near-IR dead cell stain kit (Invitrogen, Carlsbad, USA) was used to exclude dead cells from the data analysis. For GC B cell staining, cells were incubated with Brilliant Violet 421™ anti-mouse CD4 (Biolegend, clone GK1.5, San Diego, USA), FITC anti-mouse CD95(Fas) (Biolegend, clone SA367H8, San Diego, USA) and APC anti-mouse/human GL7 (Biolegend, clone GL7, San Diego, USA) for 30 minutes at 4°C. For Tfh cell staining, cells were incubated with PE/Cyanine7 anti-mouse/human CD45R/B220 (Biolegend, clone RA3-6B2, San Diego, USA), PerCP/cy5.5 anti-mouse CD185 (CXCR5) (Biolegend, clone L138D7, San Diego, USA) and PE anti-mouse CD279 (PD-1) (Biolegend, clone RMP1-14, San Diego, USA) for 30 min. After washing, cells were acquired on a BD canto II (BD Biosciences, USA), and data were analyzed in FACS Diva™ Software.

### Tetanus neurotoxin challenge of mice

Female BALB/c mice (6–8 weeks) were divided into 5 groups (16 mice per group) (**Table 1**). The five groups of mice were immunized with 1 μg MH, 1 μg Hc adjuvanted with Al, 1 μg Hc, 1 μg MT3 or PBS in final volumes of 100 μL by i.m. injection once, respectively. Fourteen days or twenty-eight days after primary immunization, eight mice from each group were challenged with tetanus neurotoxin by intraperitoneal injection of 5 LD50s of toxin with a volume of 0.4 mL in physiological solution. Mice were observed for ten days, and those alive after that time were scored as survivors.

**Table 1:** Immunization schedule and procedure.

Immunogen and Dose	Adjuvant and Dose	Route^a^	Number of mice^b^
MH, 1μg	\	i.m	16
Hc, 1μg	Al(OH)_3_, 50μg	i.m	16
Hc, 1μg	\	i.m	16
\	MT3, 1μg	i.m	16
PBS	\	i.m	16

a Immunization at 0 day (100μl/dose).

b Eight mice from each group were challenged with tetanus neutotoxin at fourteen days after immunization, the other eight mice from each group were challenged at twenty-eight days after immunization.

### Homology analysis

The pairwise and multiple alignments of MT family proteins were performed by ClustalW alignment using Geneious Prime software (2021.1.1) and BioEdit software (7.0.9.0) with default settings. For this alignment the following Uniprot/GenBank accession numbers were used: Human MT1, P04731; Human MT2, P02795; Human MT3, P25713; Human MT4, P47944; MT3 RAT, P37361; MT3 MOUSE, P28184; MT3 RABIT, Q2PS21; MT3 MACFA, Q2PFZ0; MT3 PIG, P55944; MT3 HORSE, P37360; MT3 BOVIN, P37359; MT3 BOSMU, Q2MJS5; MT3 SHEEP, Q8MKE4.

### Statistical analysis

Data were collected and presented as mean ± SEM. Statistical analysis was conducted using GraphPad v.9.0. One-way ANOVA with Tukey’s multiple comparisons tests were applied for the significant difference analysis where applicable. Survival analysis were carried out by Log-rank (Mantel-Cox) test. Statistically significant differences were considered (*p< 0.05; **p < 0.05; ***p < 0.001; ****p < 0.0001).

## Results

### The adjuvanticity of MT3 to Brucella antigen Omp19

To evaluate the adjuvant effect of human MT3 to *Brucella* antigen Omp19, MT3 was genetically fused to the N-terminus of Omp19 without signal peptide, and the fusion protein was named MT3-Omp19 (MO) (**Figure S1**).

**Figure 1:**
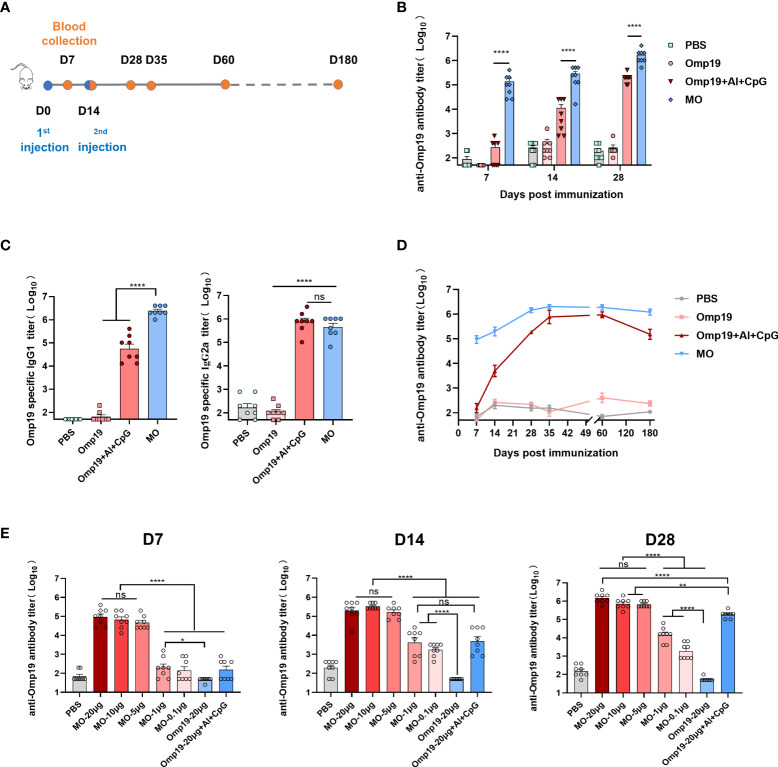
MT3 enhances the Omp19-specific antibody responses. **(A)** Immunization and blood collection schedule. Female BALB/c mice aged 6-8 week were vaccinated at day 0 and day 14 *via* i.m route with MO (20 µg), Omp19+Al+CpG (20 µg +50 µg + 25 µg), Omp19 (20 µg) or PBS (n=8). Serum samples were obtained at indicated time points for ELISA. **(B)** Omp19-specific IgG titers in sera of immunized mice. **(C)** Omp19-specific IgG subclass titers detected on day 28 in sera of immunized mice. **(D)** Kinetics of Omp19-specific total IgG titers in sera of immunized mice over a period of 180 day. **(E)** Omp19-specific IgG antibody response after immunization at day 0 and day 14 *via* i.m route with either 0.1, 1, 5, 10 or 20 μg of MO alone, Omp19+Al+CpG (20 µg +50 µg + 25 µg), Omp19 (20 µg) or PBS (n=8), respectively. Serum samples were obtained at indicated time points for ELISA. All data are presented as mean ± SEM. Statistical analyses were carried out by One-way ANOVA followed by Tukey’s multiple comparison (no significant difference, ns; *p<0.05, **p<0.005,****p<0.0001).

The immunogenicity of MO was assessed in BALB/c mice. Female mice aged 6-8 week were randomly divided into 4 groups. One group was vaccinated with 20 µg of MO on days 0, 14 by intramuscular injection (100 µL per mouse). The sera were collected on days 7, 14, 28, 35, 60, 180 to evaluate the antibody response, respectively (**Figure 1A**). The other three groups were vaccinated with 20 µg of Omp19, 20 µg of Omp19+Al+CpG or PBS as controls, respectively. The results showed that mice vaccinated with MO or Omp19 adjuvanted with Al+CpG exhibited the Omp19-specific IgG response on day 7 (**Figure 1B**), and compared to the group Al+CpG, MO induced significantly stronger specific IgG production (~343 folds higher) (1.4×10^5^ vs 4.1×10^2^, respectively). Mice administered with Omp19 without any adjuvant or PBS poorly produced serum IgG antibodies. Fourteen days after second immunization, mice vaccinated with MO produced an Omp19-specific IgG titer (1.7×10^6^) that was significantly higher than the other groups (**Figure 1B**).

To define the type of Th immune responses tuned by MT3, Omp19-specific IgG subclasses were evaluated on day 28. Compared to the group Omp19 immunized alone, MO induced significantly stronger specific IgG1 and IgG2a production. It is well-known that IgG1 and IgG2a were the hallmarks of the Th2 and Th1 pathway, respectively. The IgG1/IgG2a ratio of MO was 1.13 (>1), indicating the induction of a Th2-type immune response to MO (**Figure 1C**).

To determine whether the antibody response induced by MT3 adjuvant was persistent, IgG production in the sera of immunized mice was monitored for up to 180 days. The results showed that the MO-induced IgG response was durable, peaking after the second injection and remained high at least day 180 post-vaccination (**Figure 1D**). Compared with the Omp19 immunized alone, the antibody titer elicited by MO continuously increased by ~10^3^ folds.

To further assess the adjuvanticity of MT3, additional immunization experiments were performed by reducing the dose of injected antigens. Different immune dose groups of MO (20, 10, 5, 1, and 0.1 µg) were set. The groups immunized with 20 µg of Omp19, 20 µg Omp19 adjuvanted with Al+CpG or PBS were used as controls. Antibody titers elicited by 5 μg MO on day 7 were significantly higher (~10^3^ folds) than those of the control groups. There was no significant difference among MO 20µg, 10µg, 5µg dose groups. Fourteen days or twenty-eight days after first immunization, antibodies elicited by MO 20 µg, 10 µg, 5 µg dose groups still keep a high antibody level without significant differences. The antibodies elicited by 0.1 μg MO were higher (~10 folds) than those induced by 20 μg Omp19. This indicated that the built-in MT3 could significantly allow antigen dose sparing (**Figure 1E**).

### MT3 stimulates early antibody response and has low intrinsic immunogenicity, whose adjuvanticity relies on fusion to antigen

**Figure 2:**
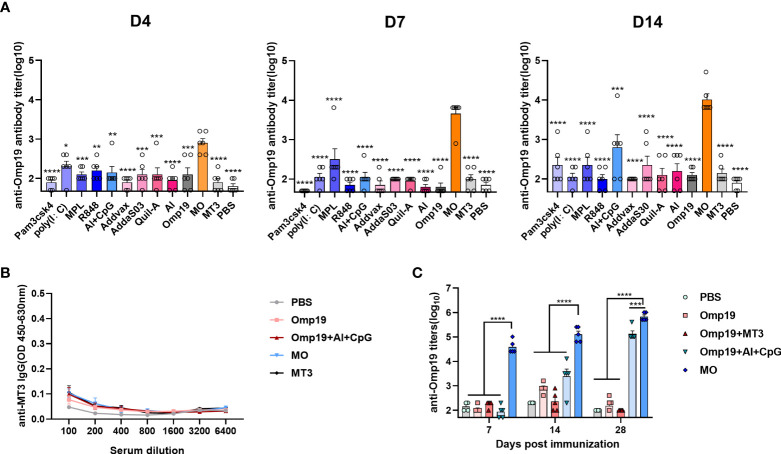
MT3 showed strong adjuvant activities with low intrinsic immunogenicity and the adjuvanticity relies on fusion to antigen. Female BALB/c mice aged 6-8 week were vaccinated at day 0 *via* i.m. route with MO (5μg), Omp19 (5μg) alone or Omp19 (5μg) combined with different adjuvants, the control group of mice were vaccinated with PBS or MT3 (n=6). Serum samples were obtained at indicated time points for measurement of Omp19-specific antibodies **(A)** and MT3-specific antibodies **(B)**. **(C)** Female BALB/c mice aged 6-8 week were vaccinated at day 0,14 *via* i.m. route with MO (5 μg), Omp19 (5μg), Omp19+MT3 (5μg+50μg), Omp19+Al+CpG (5µg+50µg+25µg) or PBS (n=5). Serum samples were obtained at indicated time points for measurement of Omp19-specific antibodies. All data are presented as mean ± SEM. Statistical analyses were carried out by One-way ANOVA followed by Tukey’s multiple comparison test (*p<0.05, **p<0.005, ***p<0.001, ****p<0.0001).

To compare the adjuvant effect of MT3 with other commercially available adjuvants, besides MO, different adjuvants were used to mix or adsorb with Omp19, and immunized mice on day 0. The Omp19-specific antibody titers on days 4, 7 and 14 were detected in mice serum. As shown in **Figure 2A**, MT3 can significantly accelerate antibody production to four days after primary immunization. In addition, MT3 induced a much stronger antibody response than all other adjuvants in the following days (~10 to100 folds). Besides, the MT3-specific antibodies in mice immunized with MO and MT3 were detected on day 14. Sera from group immunized with Omp19 adjuvanted with Al+CpG, Omp19 alone or PBS were set as controls. The result showed that MT3-specific antibody was nearly undetectable in these groups (**Figure 2B**). These suggested MT3 was a promising adjuvant with low intrinsic immunogenicity.

To test whether MT3 needs a fusion to the antigen for its adjuvanticity, antibody responses were detected in mice receiving 5 μg MO and 5 μg Omp19 mixed with 50 μg MT3, respectively. The groups immunized with 5 μg Omp19 alone, 5 μg Omp19 adjuvant with 50 μg Al + 25 μg CpG or PBS were set as controls, respectively. As shown in **Figure 2C**, the Omp19-specific antibody induced by 5 μg MO has already reached 4.6×10^4^ on day 7 and 7.2×10^5^ on day 28, which were significantly higher than the Omp19 mixed with MT3 group or Omp19 adjuvanted with Al+CpG group. Meanwhile, the Omp19 mixed with MT3 group was at the background level even after boost. The result suggested that MT3 is an effective built-in adjuvant.

### MT3 promoted the Omp19-specific DC activation, early germinal center reaction and antibody class switching

**Figure 3:**
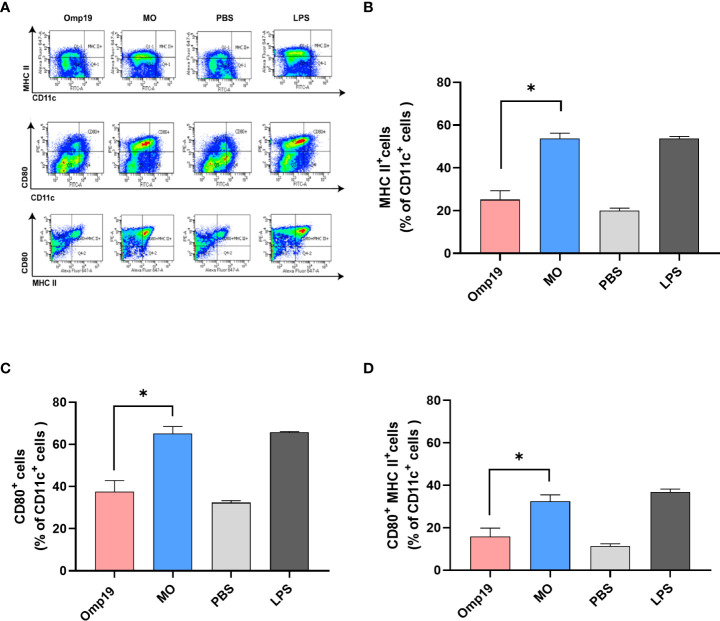
MT3 enhances the activation of Omp19-specific DCs *in vitro*. Bone marrow-derived DCs were stimulated with 10 μg/mL MO, 10 μg/mL Omp19, 0.1 μg/mL LPS or PBS for 24 h *in vitro*. **(A)** Representative flow cytometry analysis of bone marrow-derived DC. The cells were stained and the expression of CD11c/MHCII **(B)**, CD11c/CD80 **(C)** and CD11c/MHCII/CD80 **(D)** on DCs was analyzed by BD canto II, and data were analyzed in FACS Diva™ Software. All data are presented as mean ± SEM. Statistical analyses were carried out by One-way ANOVA followed by Tukey’s multiple comparison test (*p<0.05).

Dendritic cells (DCs) are important to link between innate and adaptive immunity, and activation of DCs is a vital part of initiating adaptive immune responses (22). The ability of MO to directly activate DCs was determined by analyzing the expression of MHC and co-stimulator molecules *in vitro*. BMDCs from wild type mice were treated with MO, Omp19, LPS or PBS for 24 h, respectively. As shown in **Figure 3**, following *in vitro* stimulation with MO, 53.8% CD11c^+^ MHCII^+^ DCs and 65.1% CD11c^+^ CD80^+^ DCs were observed, which were significantly higher than the Omp19-treated group (25.2% and 37.5%). Besides, 32.4% CD11c^+^ MHCII^+^ CD80^+^ DCs were also observed in the MO-treated group, which were significantly higher than Omp19 group (15.9%) or PBS group (11.3%). This suggested that incubation with MO could up-regulate the expression of T cell activation signal molecules on the surface of DCs, and promote the activation and maturation of DCs. Moreover, MO formulation may effectively induce the adaptive immunity by accelerating APC maturation.

**Figure 4:**
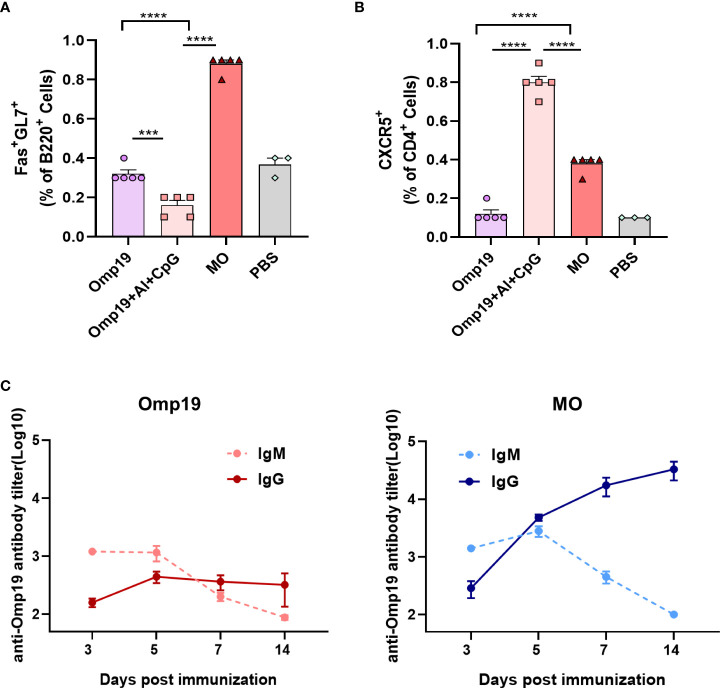
MT3 promoted Omp19-specific early germinal center reaction and antibody class switching. **(A)** Results are presented as percentage of total GC cells (B220^+^Fas^+^GL7^+^). **(B)** Results are presented as percentage of total Tfh cells (CD4^+^CXCR5^+^). **(C)** Female BALB/c mice aged 6-8 week were vaccinated at day 0 *via* i.m. route with MO (5 μg), Omp19 (5μg); Omp19-specific IgG and IgM antibodies were determined by ELISA to investigate Ig class switching (n=5). All data are presented as mean ± SEM. Statistical analyses were carried out by One-way ANOVA followed by Tukey’s multiple comparison test (***p<0.001, ****p<0.0001).

Vaccine responses are mainly generated in secondary lymphoid organs, where the antigen is presented to activated T cells and B cells within the germinal center (GC). Previously, it was observed that a high level of Omp19-specific antibody could be detected on the mouse model 4 days after MO immunization. The germinal center reaction of the proximal lymph nodes was detected at the injection site on day 4 to determine whether MO induced rapid antibody responses by promoting GC formation or not. As shown in **Figure 4A** and **Figure S3**, significant differences were observed among groups in the total GC cells (B220^+^ Fas^+^ GL7^+^ cells) in draining inguinal lymph nodes samples. Compared to mice immunized with Omp19 alone or Omp19 adjuvanted with Al+CpG group, about 0.88% GC cells were detected in MO immunized mice on day 4.

T follicular helper (Tfh) cells are a subset of CD4^+^ T cells specialized in regulating B cell responses in GCs. Total Tfh cell (CD4^+^ CXCR5^+^ cells) frequencies were evaluated on day 4. As shown in **Figure 4B** and **Figure S3**, 0.38% Tfh cells were detected in MO group, which were significantly higher than the Omp19 group. In addition, high frequencies of Tfh cells in the Omp19 adjuvanted with Al + CpG group were observed. This indicated that the Tfh cell response may begin before B cell response in GCs and prepared for the occurrence of B cell response. Moreover, these data demonstrated the early recruitment of GC B and Tfh cells *in vivo*, particularly after immunization with MO.

IgM is the earliest antibody produced by the immune system. Upon activating B cells by antigen, the immunoglobin (Ig) classes are converted from IgM to IgG. The class switching between IgM and IgG induced by the Omp19 immunized group alone occurred between 5–7 days after primary immunization (**Figure 4C**), whereas class switching induced by MO occurred 3–5 days after immunization with high IgG titers. Thus, MT3 could accelerate the Ig class switching and promote the early generation of IgG.

### Adjuvant effect of MT3 to tetanus antigen Hc

**Figure 5:**
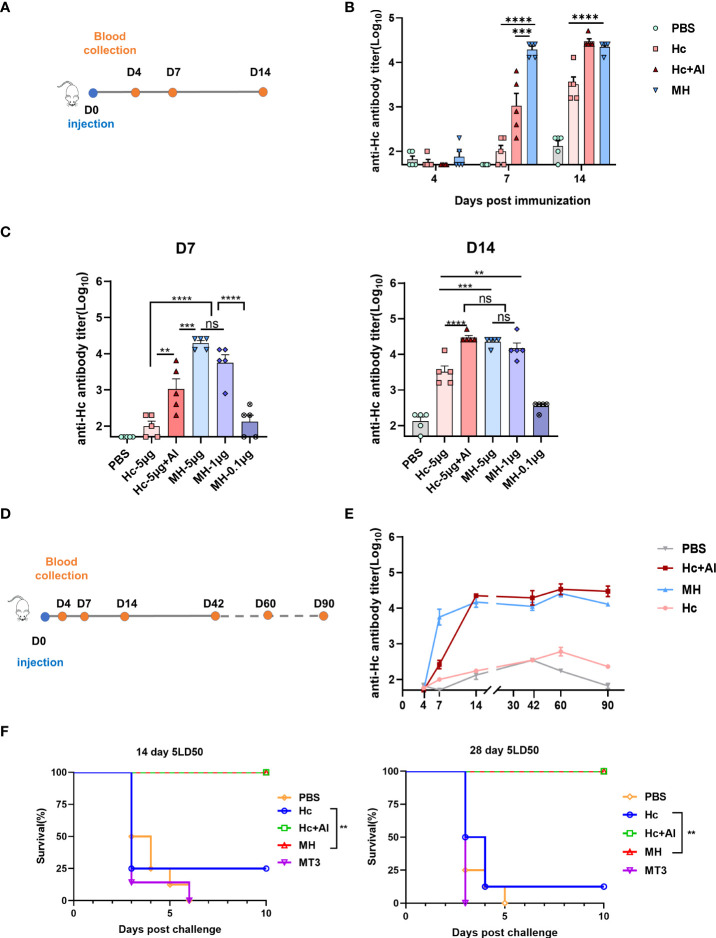
MT3 enhances the Hc-specific antibody responses. **(A)** Immunization and blood collection schedule. Female BALB/c mice aged 6-8 week were vaccinated at day 0 *via* i.m route with MH (5µg), Hc+Al (5µg+50µg), Hc (5µg) or PBS (n=5). Serum samples were obtained at indicated time points for ELISA. **(B)** Hc-specific IgG titers in sera of immunized mice. **(C)** Hc-specific IgG antibody response after immunization at day 0 *via* i.m route with either 0.1, 1, 5μg of MH alone, Hc+Al (5µg+50 µg), Hc (5µg) or PBS (n=5), respectively. Serum samples were obtained at indicated time points for ELISA. **(D)** Immunization and blood collection schedule. Female BALB/c mice aged 6-8 week were vaccinated at day 0 *via* i.m route with MH (1µg), Hc+Al (1µg +50µg), Hc (1µg) or PBS (n=5). Serum samples were obtained at indicated time points for ELISA. **(E)** Kinetics of Hc-specific total IgG titers in sera of immunized mice over a period of 90 day. All data are presented as mean ± SEM. Statistical analyses were carried out by One-way ANOVA followed by Tukey’s multiple comparison test (no significant difference, ns; **p<0.005, ***p<0.001, ****p<0.0001). **(F)** Protection against TeNT challenge. Female BALB/c mice aged 6-8 week were divided into 5 groups (16 mice per group), groups of mice were received 1μg MH, 1μg Hc adjuvant with Al, 1μg Hc, 1μg MT3 or PBS once respectively. Eight mice form each group were challenged with 5 LD50s of TeNT 14days or 28 days after vaccination. Survival was monitored every 12 h for 10 days following challenge. Statistical analyses were carried out by Log-rank (Mantel-Cox) test (**p<0.005).

Next, the adjuvant effect of MT3 was evaluated using the Tetanus antigen Hc and the fusion protein MT3-Hc (MH) was prepared (**Figure S1**). Since Hc is a protein with strong immunogenicity itself, one-shot immunization was used to investigate the immunogenicity of MH. During days 0-14, MH was observed to induce early antibody production (**Figure 5A**). Likewise, 5 μg MH elicited significantly higher antibody responses than the Hc group (~100 folds) and Hc adjuvanted with Al group (~10 folds) on day 7 (**Figure 5B**). As a built-in adjuvant, therefore, MT3 could also significantly and rapidly increase the Hc-specific antibody response.

In the dosage experiment, MT3 fusion could maintain the immune response to Hc by reduction of the antigen dosage. On day 7, the Hc-specific antibody induced by 5 μg MH was significantly higher than Hc adjuvanted with Al and Hc alone immunized group (**Figure 5C**). Significant differences were not observed between 5 μg-MH immunized group and 1 μg-MH immunized group, whereas Hc-specific antibody induced by 1 μg MH were about 100 folds higher than 5 μg Hc group. On day 14, significant differences in antibody level were not observed among the 5 μg MH group, 1 μg MH group and Hc adjuvanted with Al group. However, the antibody induced by 5 μg MH group or 1 μg MH group were significantly higher than 5 μg Hc immunized alone. In particular, the antibody titer induced by 1 μg MH were about 10 folds higher than 5 μg Hc immunized alone. The result suggested that fusion of MT3 could also allow antigen dose sparing and shorten the onset time of immune response.

Besides, Hc-specific durable immune response induced by MH was detected. As show in **Figures 5D, E**, Hc-specific antibody induced by MH still kept in a high level during 90 days, which were about 100 folds higher than the Hc immunized group persistently. The results verified that fusion of MT3 could stimulate highly effective and durable antigen-specific immune response.

The protective effect of MH was evaluated by challenge of mice. Mice were divided into 5 groups (16 mice per group), groups of mice were received 1 μg MH, 1 μg Hc adjuvant with Al, 1 μg Hc, 1 μg MT3 or PBS once, respectively. Eight mice form each group were challenged with 5 LD50s of TeNT on days 14 or 28. The result showed that MH and Hc adjuvanted with Al group could provide 100% protection (**Figure 5F**). The protection provided by Hc immunized group was only 25% on day 14 or 12.5% on day 28. The mice immunized with MT3 or PBS were all dead in 6 days. The survival rate of the MH-immunized group was significantly higher than that of the Hc-immunized group alone. Thus, MT3 could effectively increase the immune protection of Hc.

### Homology analysis and immunogenicity study of MT family proteins

**Figure 6:**
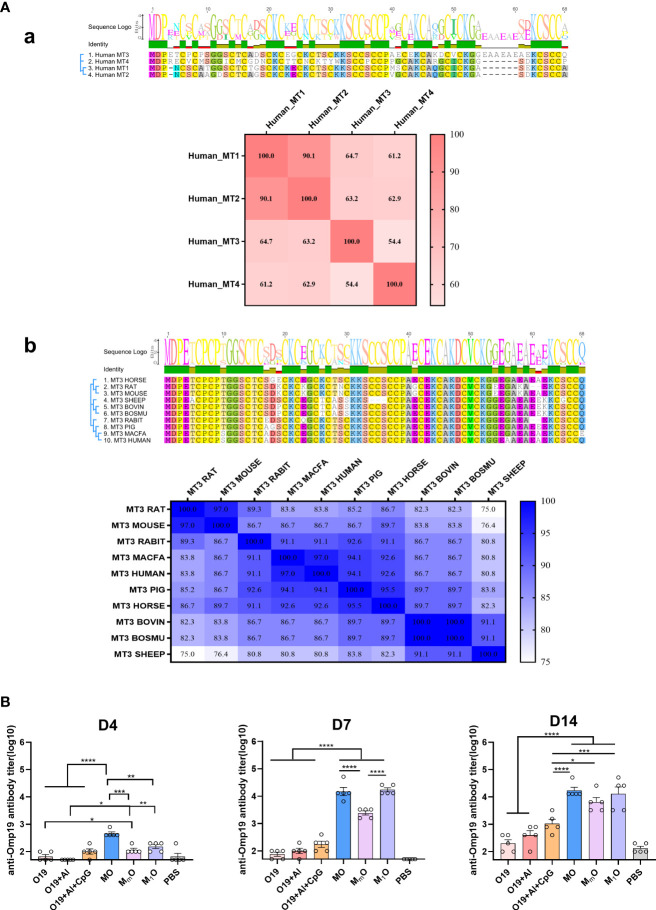
Homology analysis and immunogenicity study of MT family proteins. **(A)** Sequence alignment of MTs family proteins. **(a)** Amino acid sequence alignment between MT3 and other human-derived MT family proteins. **(b)** Amino acid sequence alignment between human MT3 and other species-derived MT3 proteins. Sequence alignment was prepared with Geneious Prime (2021.1.1) and BioEdit software (7.0.9.0). **(B)** Immunogenicity study of MT family proteins. Female BALB/c mice aged 6-8 week were vaccinated at day 0 *via* i.m route with MO (5µg), M_m_O (5µg), M_1_O (5µg), Omp19 (5µg), Omp19+Al (5µg +50µg), Omp19+Al+CpG (5µg +50 µg+25µg) or PBS (n=5). Serum samples were obtained at indicated time points for measurement of Omp19-specific antibodies. All data are presented as mean ± SEM. Statistical analyses were carried out by One-way ANOVA followed by Tukey’s multiple comparison test (*p<0.05, **p<0.005, ***p<0.001, ****p<0.0001).

The representative sequences of mammalian MT family were compared by Geneious Prime software (2021.1.1) and BioEdit software (7.0.9.0). The homology between multiple sequences of human MTs and MT3 sequences from multiple species was compared. Among the four human MT subtypes, the homology values of MT1-MT2, MT1-MT3, MT1-MT4 and MT3-MT4 were 90.1%, 64.7%, 61.2% and 54.4%, respectively (**Figure 6A**). Moreover, the homology of MT3 from common species such as rat (*Rattus norvegicus*), mouse (*Mus musculus*), rabbit (*Oryctolagus cuniculus*), macfa (*Macaca fascicularis*), pig (*Sus scrofa*), horse (*Equus caballus*), bovin (*Bos taurus*), sheep (*Ovis aries*), bosmu (*Bos mutus grunniens*) and human (*Homo sapiens*) were also compared, the result show the homology in all the species were nearly above 80%, except sheep compared to rats (75%) or mice (76.4%).

To explore whether other MT proteins have adjuvant effects or not, human MT1 and murine MT3 were selected as representatives to fused with Omp19, and the fusion proteins MT1(H)-Omp19 (M_1_O) and MT3(M)-Omp19 (M_m_O) were prepared (**Figure S2**). The results showed that human MT3 could help to induce significantly higher antigen-specific antibody titer than human MT1 and murine MT3 four days after primary immunization. Seven days later, the specific antibody titer stimulated by human MT1 based protein (M_1_O) increased significantly. The antigen specific antibody titer stimulated by human MT1 and MT3 were significantly higher than murine MT3. Fourteen days after primary immunization, the antibody titer induced by MT-based proteins were still higher than that of Omp19 immunized alone, Omp19 with Al or Omp19 with Al + CpG group (**Figure 6B**). Thus, the mammalian MTs family (human MT1 or murine MT3 for examples) also had potential adjuvanticity, although their effects were lower than human MT3.

## Discussion

As an important component, adjuvants can significantly increase the effectiveness of vaccines, especially recombinant subunit vaccines. The ideal vaccine adjuvants should be able to magnify the immunogenicity of weak antigens, accelerate and prolong the desired immune responses, allow required antigen dose sparing, reduce the frequency of immunization, broaden the breath of immune response and improve the effectiveness of the vaccine for those who do not respond well. These were also the crucial gaps in modern vaccine product development need to be filled by appropriate adjuvant technologies (1).

In this study, MT3 was studied as a novel built-in vaccine adjuvant for recombinant protein vaccines. When it was fused with Omp19, the fusion protein MO increased the antibody titers by approximately 340 folds compared to the Omp19 adjuvanted with Al+CpG, or 1000 folds compared to the Omp19 alone in seven days after primary immunization. When immunized twice (days 0 and 14), the antibody titers induced by MO persisted at a high level until day 180, suggesting that the MT3 could help to maintain a durable antigen-specific immune responses. Results from the dose research showed that the antibody titers elicited by 5 μg MO increased by about 1000 folds compared to 20 μg Omp19 immunized alone on day 7. And the antibody response elicited by 0.1 μg MO was 10 folds higher than 20 μg Omp19 on day 14. It suggested that MT3 may help to achieving antigen dose sparing. It is well known that the production ability inhibits the global vaccine supply. If an adjuvant could remarkably reduce the antigen usage in a vaccine for induction of target antibody titers, it will increase the global vaccine supply and save more people around the world (23).

When Hc was used as a model antigen, similar to the results observed in MO, MT3 could help the fusion protein MH to stimulate a rapid, highly effective, durable Hc-specific immune response, and reduced acquired antigen dose. It was observed that the antibody titers elicited by MH were slightly lower than that of Hc adjuvanted with Al 14 days after immunization, the possible reason may be the strong immunogenicity of Hc itself, but the adjuvant effect of MT3 was still obvious. Besides, MH led to the full protection of the immunized mice against a lethal dose tetanus toxin challenge, suggested that MT3 could increase the neutralization antibody titers as well as the overall antigen-specific antibodies.

Moreover, the adjuvant capacity of MT3 was compared with several commercially available adjuvants. The adjuvants included the broadly used aluminum hydroxide adjuvant, TLR-based adjuvants such as Pam3CSK4 (TLR1/2 agonist) (24), polyI:C (TLR3 agonist) (25, 26), MPLA-SM (TLR4 agonist) (27), R848 (TLR7/8 agonist) (28), CpG (TLR9 agonist) (28), and squalene-based oil-in-water adjuvant AddaVax (based on the formulation of MF59) (29), AS03-like vaccine adjuvant (AddsS03) (30), and saponin vaccine adjuvant (Quil-A) (1). As a built-in adjuvant, MT3 could dramatically accelerate the antibody production up to four days after primary immunization, and the antibody titers significant increased by 10 to 100 folds in comparison to other adjuvants. The results demonstrated a rapid and robust immune response induced by MT3 again.

In order to explore the mechanism of MT3 enhancing early acquired immune response, several key points were chosen for evaluation. DC is the most efficient antigen presentation cell (APC) and is the only APC that can activate the primary T-cells. Consequently, DC is often considered to be the initiator of the adaptive immune response. In general, most DCs are immature with low antigen presentation capacity (31, 32). They express low levels of MHC-II molecules and costimulatory molecules (such as CD40, CD80 and CD86). However, immature DCs express FcR and pattern recognition receptors (such as mannose receptors, toll like receptors, etc.) at high levels, and have strong antigen uptake and processing capabilities (33). After ingesting the antigen or certain stimulation, immature DCs begin to differentiate and mature, which is typically characterized by a significant up-regulation in the expression level of MHC-II molecules and costimulatory molecules, effectively activating T cells and initiating immune responses (34). In our study, incubation of MO with BMDC significantly up-regulated MHC-II and CD80 level on the surface of DCs. When MO was ingested by DC *in vivo*, more MHC-II molecular were expressed to present antigen peptide to T cell due to the stimulation of MT3, and the CD80 was up-regulated as a second activation signal to activate T cell. Then, T cell began to proliferate and differentiate to produce CD4^+^ Th cells and Tfh cells.

The process of B cells differentiating into plasma cells is incredibly vital to production of antibodies in adaptive response. With the help of Tfh cell, the activated B cell moved to the follicular area for proliferation, and at the absence of T cell to create a unique structure (the germinal center, GC) (35). B cells manufacture antibodies with high affinity and numerous subtypes when undergoing somatic hypermutation (SHM) and class switch reaction (CRS) in GC (36, 37). GC is the key to the production of high affinity antibodies and the main supply of long-lived plasma cells, which also makes the antibody in the body persist (38). In our study, high level of antibody titer elicited by MO was observed as early as day 4 in mice model, and the GC reaction of the proximal lymph nodes was thus detected at the injection site on day 4. The frequencies of GC B cell obviously increased and the high frequencies of Tfh cell were elicited. The maturation of B cells to plasma cells can occur in three main ways (39). The way that plasma cells are generated from germinal center B cells in the course of follicular responses is the most concerned way, and in this way, the time for formation of germinal center is regarded to be 6-7 days at least (35). Our results seemed not consistent with the conventional views, one explanation is that the observed antibody response on day 4 may be contributed by both GC reaction and extrafollicular response, although the detailed mechanism needs further study.

Considering the sequence and structure similarity of mammalian MT family proteins, we selected human MT1 and murine MT3 as representatives to explore whether other MT proteins have adjuvant effects. The results showed that both human MT1 and murine MT3 could enhance immune response to fusion proteins M_1_O and M_m_O, but human MT3 could help to induce a more rapid antigen specific immune response than human MT1 and murine MT3. And human MT1/MT3 could help to induce a higher immune response than murine MT3. Thus, the MT family members also had potential adjuvanticity, but the difference between species and the amino acid sequence consistency could affect the adjuvant effect of MTs. According to our results, it is difficult to compare the adjuvant effects of murine MT3 or human MT3 in human, but as a potential adjuvant for human vaccines, the human MT3 may be the better choice, and the further safety evaluation is needed.

At present, the molecular mechanisms of adjuvant activity of MT3 are not clear yet. As a member of MTs, MT3 could regulate the homeostasis of heavy metal such as copper and zinc (Zn). Zn homeostasis is essential to all biological processes, and it was reported that Zn deficiency or excess is not conducive to antibody and cytokine production, proliferation and function of immune cells (7). On the other hand, MT3 mainly expresses in the central nervous system, and it was reported that brain activity may directly control the adaptive immune response of lymphoid organs (40, 41). Whether MT3 regulates adaptive immune response by modulating Zn homeostasis and the participation of its neuron inhibitor function are worthy of further investigation.

In conclusion, this study provided convincing evidence that human MT3 as a novel built-in adjuvant can help protein antigens to stimulate rapid, effective, and durable antibody responses, which may have important clinical application potential in vaccine development against global pandemics.

## Data availability statement

The original contributions presented in the study are included in the article/**Supplementary Material**. Further inquiries can be directed to the corresponding authors.

## Ethics statement

The animal study was reviewed and approved by the Laboratory Animal Care and Use Committee of the Beijing Institute of Biotechnology.

## Author contributions

WC, JX, and YY were responsible for overall experimental design and supervision of studies. YG, YY, RL, RY, SW and YZ performed experiments. XdZ and YY performed bioinformatics analysis. YY, YG, XdZ and RL analyzed the data. XjZ, JL, and JZ contributed reagents and materials. YY, JX and YG wrote the manuscript. WC, JX, and JL reviewed the manuscript. All authors contributed to the article and approved the submitted version.

## Funding

This work was partially supported by the National Natural Science Foundation of China (32170945).

## Acknowledgments

We thank Dr. Tao Hu for useful discussion and language editing. We thank Yujie Li and Jianglan Long for technical assistance. We thank Dr. Yilong Yang, Dr. Yaohui Li and Dr. Chuge Zhou for helpful comments on the manuscript.

## Conflict of interest

The authors declare that the research was conducted in the absence of any commercial or financial relationships that could be construed as a potential conflict of interest. Beijing Institute of Biotechnology has submitted patent applications related to use of MTs as vaccine adjuvants.

## Publisher’s note

All claims expressed in this article are solely those of the authors and do not necessarily represent those of their affiliated organizations, or those of the publisher, the editors and the reviewers. Any product that may be evaluated in this article, or claim that may be made by its manufacturer, is not guaranteed or endorsed by the publisher.

## Supplementary material

Supplementary Figure 1 Characterization and analysis of the MT3-based proteins. **(A)** MT3 based structure design. **(B)** SDS-PAGE analysis of the purified MT3-based proteins. **(C)** Analysis of the purified MT3-based proteins by Western blot. The bands were probed with Omp19 immunized serum against Omp19 and MO, with human monoclonal antibodies against Hc and MH.

Supplementary Figure 2 Characterization and analysis of MT-based proteins. **(A)** MT based structure design. **(B)** SDS-PAGE analysis of the purified MT-based proteins. **(C)** Analysis of the purified MT-based proteins by Western blot. The bands were probed with Omp19 immunized serum against Omp19, M_m_O, and M_1_O.

Supplementary Figure 3 Representative flow cytometry analysis of GC B cells, Th cells at day 4. The cells were stained and the expression of B220^+^Fas^+^GL7^+^ (GC B cells) and CD4^+^CXCR5^+^ (Tfh cells) was analyzed by BD canto II, and data were analyzed in FACS Diva™ Software.
