# Supplementary figures and images for: A novel built-in adjuvant metallothionein-3 aids protein antigens to induce rapid, robust, and durable immune responses
Source: Front Immunol. 2022 Nov 8;13:1024437. doi: 10.3389/fimmu.2022.1024437 (PMC9680554; doi:10.3389/fimmu.2022.1024437)

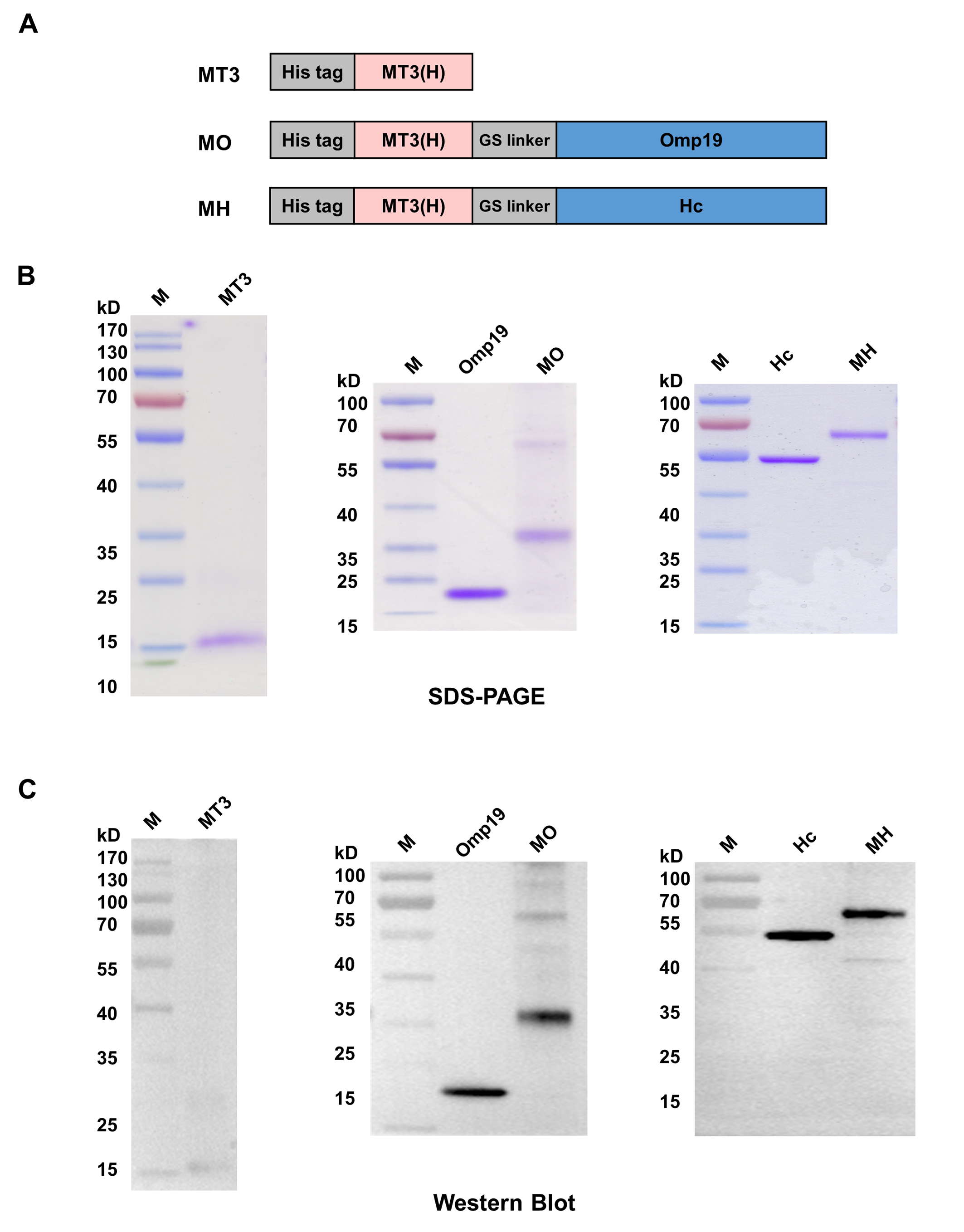

Supplement: Supplementary Figure 1 — Characterization and analysis of the MT3-based proteins. (A) MT3 based structure design. (B) SDS-PAGE analysis of the purified MT3-based proteins. (C) Analysis of the purified MT3-based proteins by Western blot. The bands were probed with Omp19 immunized serum against Omp19 and MO, with human monoclonal antibodies against Hc and MH. [file Image_1.tif]

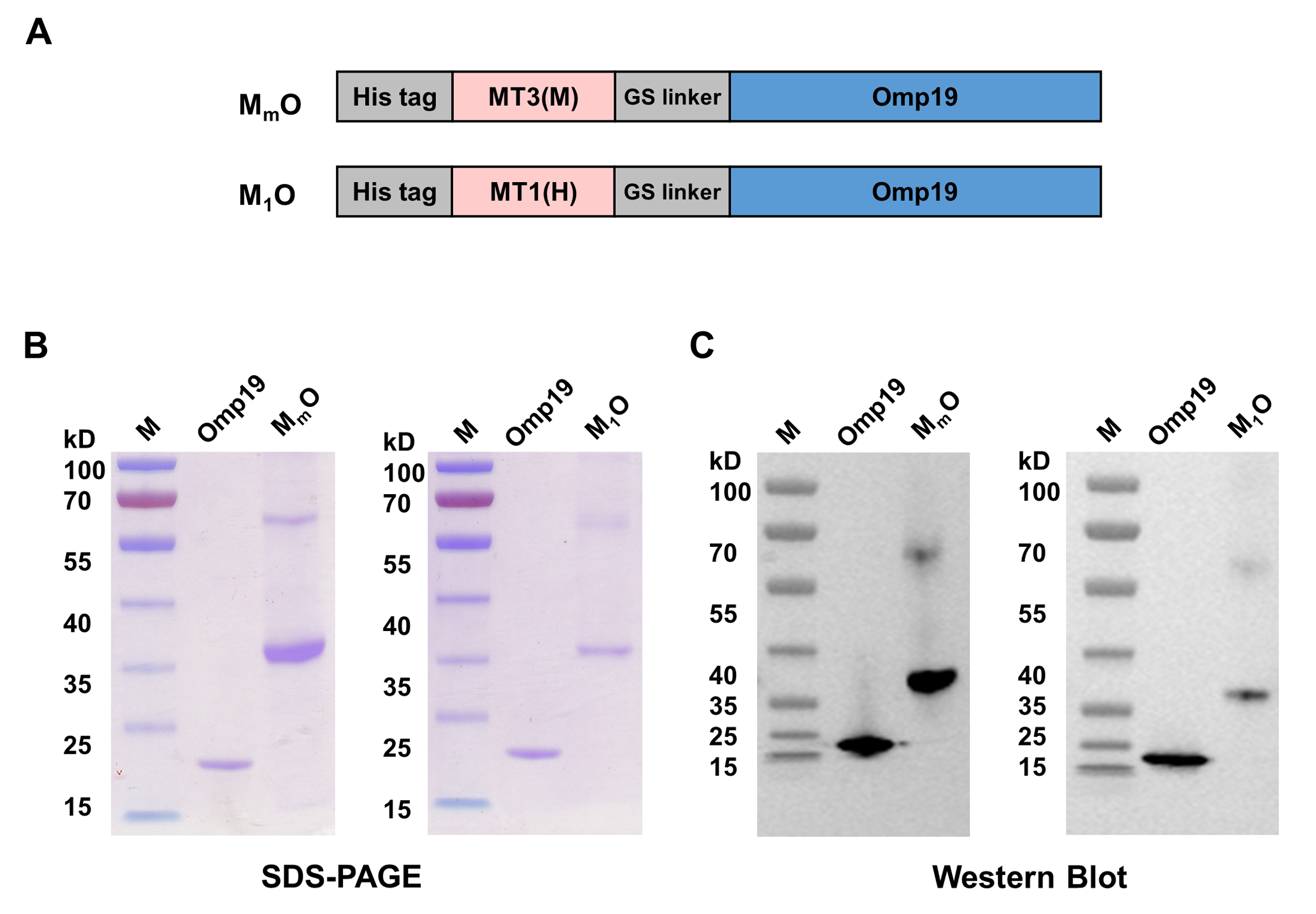

Supplement: Supplementary Figure 2 — Characterization and analysis of MT-based proteins. (A) MT based structure design. (B) SDS-PAGE analysis of the purified MT-based proteins. (C) Analysis of the purified MT-based proteins by Western blot. The bands were probed with Omp19 immunized serum against Omp19, MmO, and M1O. [file Image_2.tif]

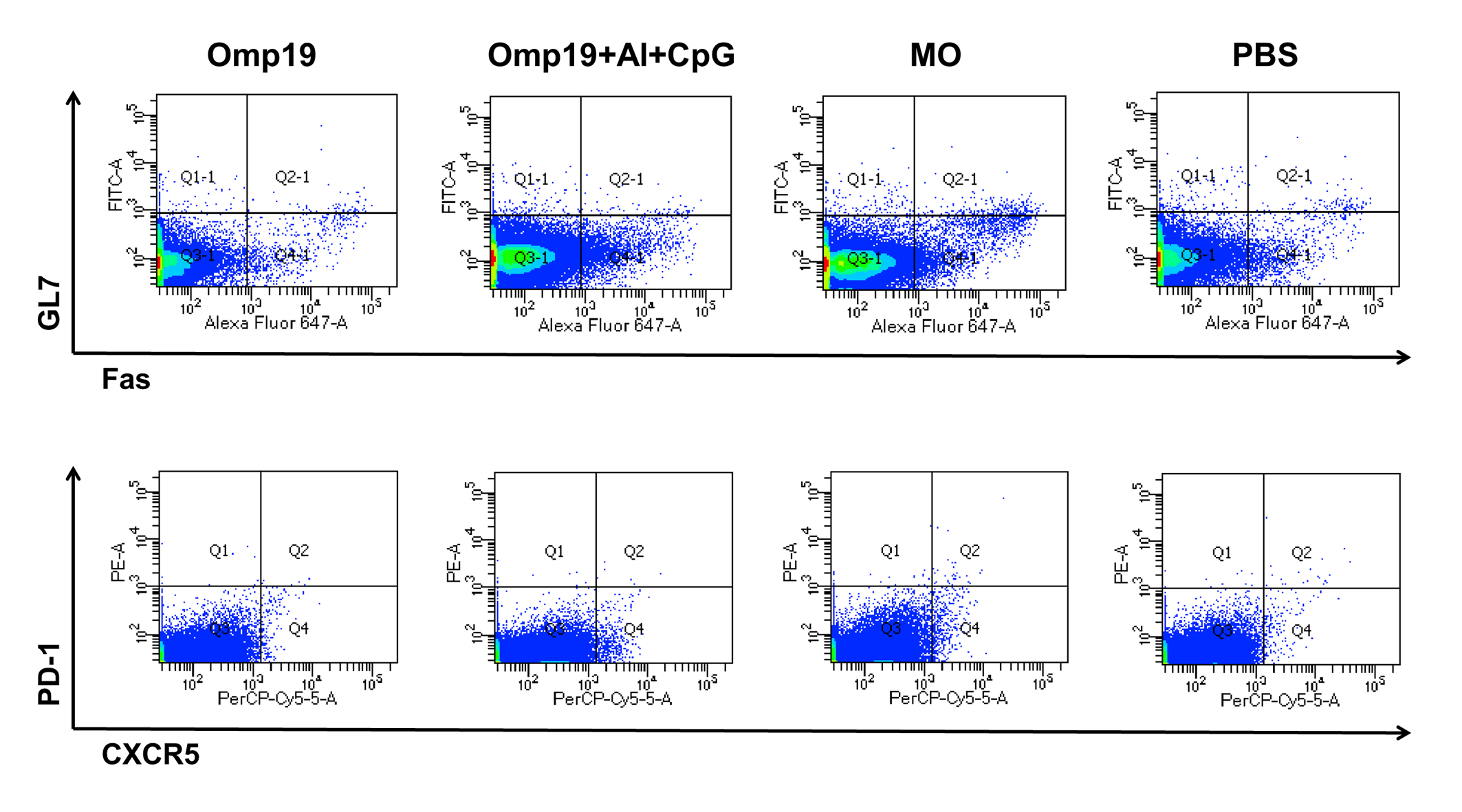

Supplement: Supplementary Figure 3 — Representative flow cytometry analysis of GC B cells, Th cells at day 4. The cells were stained and the expression of B220+Fas+GL7+ (GC B cells) and CD4+CXCR5+ (Tfh cells) was analyzed by BD canto II, and data were analyzed in FACS Diva™ Software. [file Image_3.tif]
